# Post-stroke upper limb recovery is correlated with dynamic resting-state network connectivity

**DOI:** 10.1093/braincomms/fcae011

**Published:** 2024-01-23

**Authors:** Chih-Wei Tang, Catharina Zich, Andrew J Quinn, Mark W Woolrich, Shih-Pin Hsu, Chi-Hung Juan, I Hui Lee, Charlotte J Stagg

**Affiliations:** Institute of Brain Science, Brain Research Center, National Yang Ming Chiao Tung University, Taipei City 112, Taiwan; Department of Neurology, Far Eastern Memorial Hospital, New Taipei City 220, Taiwan; Nuffield Department of Clinical Neurosciences, Wellcome Centre for Integrative Neuroimaging, FMRIB, University of Oxford, Oxford OX3 9DU, UK; MRC Brain Network Dynamics Unit, University of Oxford, Oxford, OX1 3TH, UK; Nuffield Department of Clinical Neurosciences, Wellcome Centre for Integrative Neuroimaging, FMRIB, University of Oxford, Oxford OX3 9DU, UK; Department of Psychiatry, Oxford Centre for Human Brain Activity, Wellcome Centre for Integrative Neuroimaging, University of Oxford, Oxford OX3 7JX, UK; Centre for Human Brain Health, School of Psychology, University of Birmingham, Birmingham B15 2TT, UK; Nuffield Department of Clinical Neurosciences, Wellcome Centre for Integrative Neuroimaging, FMRIB, University of Oxford, Oxford OX3 9DU, UK; Department of Psychiatry, Oxford Centre for Human Brain Activity, Wellcome Centre for Integrative Neuroimaging, University of Oxford, Oxford OX3 7JX, UK; Institute of Brain Science, Brain Research Center, National Yang Ming Chiao Tung University, Taipei City 112, Taiwan; Institute of Cognitive Neuroscience, National Central University, Taoyuan City 320, Taiwan; Institute of Brain Science, Brain Research Center, National Yang Ming Chiao Tung University, Taipei City 112, Taiwan; Division of Cerebrovascular Diseases, Neurological Institute, Taipei Veterans General Hospital, Taipei City 112, Taiwan; Nuffield Department of Clinical Neurosciences, Wellcome Centre for Integrative Neuroimaging, FMRIB, University of Oxford, Oxford OX3 9DU, UK; MRC Brain Network Dynamics Unit, University of Oxford, Oxford, OX1 3TH, UK

**Keywords:** stroke, motor recovery, magnetoencephalography, hidden Markov model

## Abstract

Motor recovery is still limited for people with stroke especially those with greater functional impairments. In order to improve outcome, we need to understand more about the mechanisms underpinning recovery. Task-unbiased, blood flow–independent post-stroke neural activity can be acquired from resting brain electrophysiological recordings and offers substantial promise to investigate physiological mechanisms, but behaviourally relevant features of resting-state sensorimotor network dynamics have not yet been identified. Thirty-seven people with subcortical ischaemic stroke and unilateral hand paresis of any degree were longitudinally evaluated at 3 weeks (early subacute) and 12 weeks (late subacute) after stroke. Resting-state magnetoencephalography and clinical scores of motor function were recorded and compared with matched controls. Magnetoencephalography data were decomposed using a data-driven hidden Markov model into 10 time-varying resting-state networks. People with stroke showed statistically significantly improved Action Research Arm Test and Fugl-Meyer upper extremity scores between 3 weeks and 12 weeks after stroke (both *P* < 0.001). Hidden Markov model analysis revealed a primarily alpha-band ipsilesional resting-state sensorimotor network which had a significantly increased life-time (the average time elapsed between entering and exiting the network) and fractional occupancy (the occupied percentage among all networks) at 3 weeks after stroke when compared with controls. The life-time of the ipsilesional resting-state sensorimotor network positively correlated with concurrent motor scores in people with stroke who had not fully recovered. Specifically, this relationship was observed only in ipsilesional rather in contralesional sensorimotor network, default mode network or visual network. The ipsilesional sensorimotor network metrics were not significantly different from controls at 12 weeks after stroke. The increased recruitment of alpha-band ipsilesional resting-state sensorimotor network at subacute stroke served as functionally correlated biomarkers exclusively in people with stroke with not fully recovered hand paresis, plausibly reflecting functional motor recovery processes.

## Teaser

Tang *et al*. report that resting electrophysiological data give important insights into stroke recovery. Magnetoencephalography data-driven analysis from 37 people with stroke and 22 controls showed the dynamics of the alpha-band ipsilesional sensorimotor network altered at 3 weeks after stroke and the network dynamics metrics correlated with functional motor score.

## Graphical Abstract

**Graphical Abstract:**
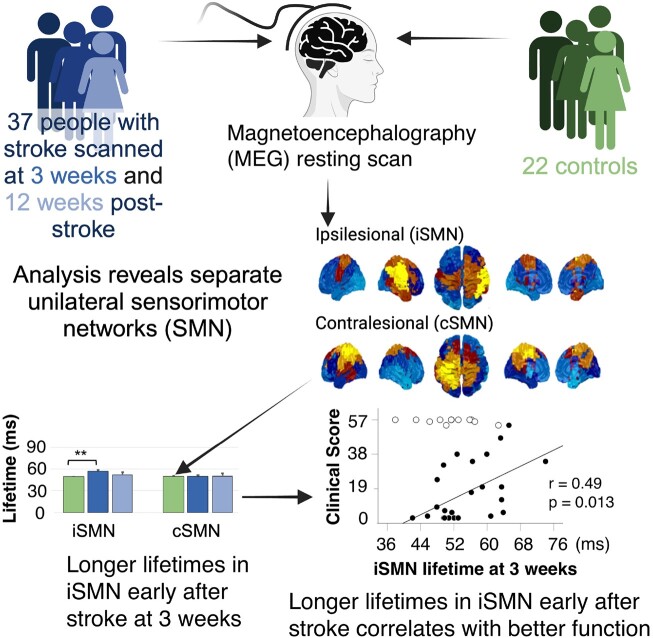

## Introduction

Motor recovery after stroke is still suboptimal for most people and particularly for those with moderate to severe hand paresis.^1-3^ Insights into the mechanisms underlying recovery could significantly advance the development of potential therapeutic options. While upper limb recovery can be predicted by initial motor impairment using the proportional recovery model in patients with less severe hand paresis, this method often fails for severely impaired patients.^4^ Studying changes in neural activity is therefore important in order to understand the mechanisms of motor recovery and to then harness these to improve outcome. However, most task-based studies excluded people with stroke with poor hand motor function as they are unable to perform the motor tasks adequately. Resting-state biomarkers are unbiased by motor task performance and have the potential to disclose the post-stroke motor recovery processes independent of motor impairment. The motor evoked potential (MEP) induced by transcranial magnetic stimulation at rest can provide supplementary information in predicting motor outcome,^5^ but it’s reliability is still debated.^6^ Resting-state functional MRI (rs-fMRI) studies have demonstrated decreased functional connectivity of the sensorimotor network (SMN) early after stroke, which then increased with recovery of motor function.^7^ However, fMRI is dependent on the blood oxygen level–dependent (BOLD) signal, which may be unreliable post-stroke due to impaired vasoreactivity.^8^

Resting neural activity can be easily acquired using magneto- or electro-encephalography (MEG/EEG), which can give an insight into temporal, spectral and spatial features of neural activity, such as spectral power or connectivity, which may underlie recovery.^9-13^ Previous studies have shown increased power in low-frequency activity in the perilesional region early after stroke compared with controls,^11^ which decreases during recovery.^10^ Alpha coherence has also been noted to increase in perilesional regions after stroke, and the degree of coherence predicts motor outcome after controlling for age, stroke onset time and lesion size in voxel-wised coherence study.^12^ However, these previous studies have investigated changes in neural activity within pre-defined frequency bands of interest and have not investigated temporally varying neural activity features, which are increasingly understood to be functionally relevant.^9-12^

Hidden Markov models (HMM) enable the decomposition of electrophysiological data into discrete functional states with millisecond precision.^14-16^ The HMM estimates brain states in a data-driven manner without pre-specification or constraining the timescale of dynamics and can be applied to resting^17,18^ or task^17,19^ data. The dynamic features of each brain state, such as interval time (i.e. time between two state visits) or life-time (i.e. time of a state visit), can be quantified. HMM has been successfully used in a number of applications, including the study of memory replay^20^ and in neurological diseases, including dementia and multiple sclerosis,^18,21,22^ where the different metrics are able to give more specific insights into aspects of function. For example, the ability to switch between brain states, inversely reflected by the how long the brain spends in a given state at a time (the ‘life-time’), has been suggested to be important in flexible cognitive control, where rapid shifting may reflect faster behavioural responses.^14,23^ The amount of time the brain spends in a given state (the ‘fractional occupancy’ or FO) is closer to the classic spectral ‘power’ of a standard, temporally invariate, analysis.^23^ We therefore predict that people with stroke will have a low-frequency (delta to alpha) ipsilesional SMN (iSMN), which will exhibit longer life-times and greater FO than age-matched control participants.

Here, we conducted a longitudinal MEG study in a cohort of people with stroke with first-time subcortical infarction in the middle cerebral artery (MCA) territory at Weeks 3 (early subacute stage) and 12 (late subacute stage)^24^ after stroke with different degrees of hand paresis to test a number of hypotheses: (i) subacute stroke is characterized by specific temporal features of ipsilesional resting-state SMN, including longer life-times and greater FO. (ii) The resting-state SMN changes correlate with stroke severity and functional outcome. (iii) SMNs estimated from resting-state data and applied to motor task data yield clinically relevant information.

## Materials and methods

MEG recordings of people with stroke and age- and sex-matched controls were collected from the MEG database established at Taipei Veterans General Hospital, Taiwan, from 2009 to 2018. Inclusion criteria were age 20–80, right-handed as determined by Edinburgh Handedness Inventory^25^ and, for people with stroke, first-ever, unilateral, subcortical ischaemic stroke in the MCA territory, with hand weakness of any degree, 2–4 weeks after stroke. Exclusion criteria were concomitant major neurological diseases or severe medical diseases. The study was approved by the ethics committee of the Taipei Veterans General Hospital (2013-06-036B, 2014-01-006C, 2015-03-003C), with written informed consent obtained from each participant. The study adhered to the Declaration of Helsinki, except for preregistration. As there were no similar studies on which to base power calculations, all eligible people with stroke who consented within the recruitment period were included.

People with stroke attended at two timepoints: at 3 weeks post-stroke (range 2–4 weeks) as baseline and at 12 weeks post-stroke (range 11–13 weeks). At each session, MEG data (see below) were recorded, and the Fugl-Meyer upper extremity (FM-UE) score^26^ and the Action Research Arm Test (ARAT) score^27^ were conducted by a clinical therapist blinded to the study design. The identical MEG protocol was performed once in controls. In addition, MRI data were acquired once (see below).

### MEG acquisition

MEG data were recorded in a magnetically shielded room using a whole-head array 306-channel Neuromag Vectorview^TM^ MEG system (Elekta, Helsinki, Finland) with a 500 Hz sampling rate. Head position indicator (HPI) coils were placed at four locations on the head to record head position relative to the MEG sensors. Head landmarks (pre-auricular points and nasion) and 30–40 points on the scalp, face and nose were digitized using a Polhemus Isotrak II system. Electrooculography channels over the right upper and left lower orbital regions and electrocardiography channels over both arms were recorded (500 Hz) to detect blink, ocular and cardiac artefacts.

Resting-state MEG was acquired for 5 min, with the subject seated, eyes open and body relaxed. The motor task paradigm was a simple motor task (see Woolrich *et al.*^28^ for details). Briefly, subjects performed a self-paced unilateral index finger lifting task every 7 s for paretic (left in controls) hand then non-paretic (right in controls) hand consecutively. A total of 100 trials were collected for each hand, with a short break after the first 50 trials (total duration ∼20 min). The movement onset of finger lifting was detected by an optical detection pad.

### MRI acquisition and analysis

All subjects underwent 3-Tesla MRI scan (Discovery MR750, General Electric Company). This included high-resolution three-dimensional T_1_-weighted structural images (repetition time, TR = 12.2 ms; echo time, TE = 5.2 ms; flip angle = 12°; voxel size = 1 × 1 × 1 mm; field of view, FoV = 256 × 256 mm) and diffusion-weighted imaging (TR = 4060 ms, TE = 64 ms, thickness = 5 mm, *b* = 1000 s mm^−2^). Lesion volumes were manually delineated from the diffusion-weighted images by an experienced vascular neurologist then plotted to standard space using MRIcron®. To produce group lesion maps, heatmaps of ischaemic stroke lesions were generated for right and left hemispheric strokes separately.

### MEG analysis

The resting-state MEG raw data were initially processed by spatiotemporal signal space separation (TSSS) correction implemented in MaxFilter version 2.2.10 (Elekta Neuromag, Elekta, Stockholm, Sweden) to reduce external noise. Further MEG analysis was performed using the OHBA Software Library (OSL: https://ohba-analysis.github.io/osl-docs/) version 2.2.0.

Pre-processing steps were applied prior to HMM analysis, as detailed by Quinn *et al*. and briefly outlined here. First, the structural MRI and the MEG data were co-registered using Registration of Head shapes Including Nose in OSL (RHINO). Continuous MEG data were then down-sampled to 250 Hz to reduce computational demands. Independent component analysis (ICA) was used to estimate 64 independent components using temporal FastICA. Independent components representing stereotypical artefacts such as eye blinks, eye movements and electrical heartbeat activity were manually identified and regressed out of the data. Cleaned data were then band-pass filtered (1–40 Hz) and projected onto an 8 mm grid in source space using a linearly constrained minimum variance (LCMV) vector beamformer.^29^ A weighted (non-binary) parcellation with 39 cortical regions was applied, and parcel-wise time-courses were estimated following the methods of Colclough *et al.*^30^ Symmetric orthogonalization was applied to each data set prior to concatenation to remove spatial leakage. Resting-state data were analysed as continuous data (300 s). For the motor task, data were epoched from −3 to 3 s relative to the movement onset.

We inferred the time delay embedded HMM (TDE-HMM) for all 39 cortical regions, which allows for identification of states with distinct multi-region spectra and/or phase locking networks. The resting-state data from people with stroke and matched controls were temporally concatenated over subjects prior to HMM. Then, source-reconstructed time-courses for each parcel were time delay embedded using 15 lags corresponding to time indexes between −7 and 7, hence specifying a 30 ms lag in both directions at the sampling rate of 250 Hz. As 10 HMM states yield 1 lesion side–specific SMN, we opt for 10 HMM states in this study.

We then extracted temporal metrics that summarize the dynamics of each HMM state, including life-time, interval time and FO. Life-time (or dwell-time) of each state is defined as the average time elapsed between entering and exiting a state. Interval length is defined as the time elapsed between visits to a state. FO is defined as the proportion of time spent in each state.^23^

Lastly, to assess whether the results inferred from HMM states during resting state are generalizable to a motor context, we used the HMM state descriptions estimated from the resting state to investigate dynamics during motor activation. This approach allows to investigate the same properties of the data, rather than global changes, and has been used previously to examine effects of transfer or generalizability.^18,31^ The temporal metrics (life-time, interval time and FO) of these resting brain states during paretic (or left in controls) hand movement and non-paretic (or right in controls) were then quantified.

### Statistical analysis

All analyses on HMM parameters and behavioural data were performed using the SPSS software (version 25, Chicago, IL, USA). Demographic characteristics between the stroke and the control group were examined using chi-square tests for categorical data and Mann–Whitney U tests for continuous data. Multivariate analysis of variance (ANOVA)s were performed to compare the HMM parameters (life-time, interval time, FO) between control and stroke groups. To evaluate the changes in HMM parameters over time, one-way repeated measures ANOVAs were performed. Significant effects in the ANOVAs were followed by *post hoc* Mann–Whitney U test (independent data) or Wilcoxon signed rank test (dependent data) with Bonferroni correction where appropriate. The relationship between motor scores and HMM parameters was examined using Spearman’s rank correlation.

## Results

### Demographics

**Figure 1:**
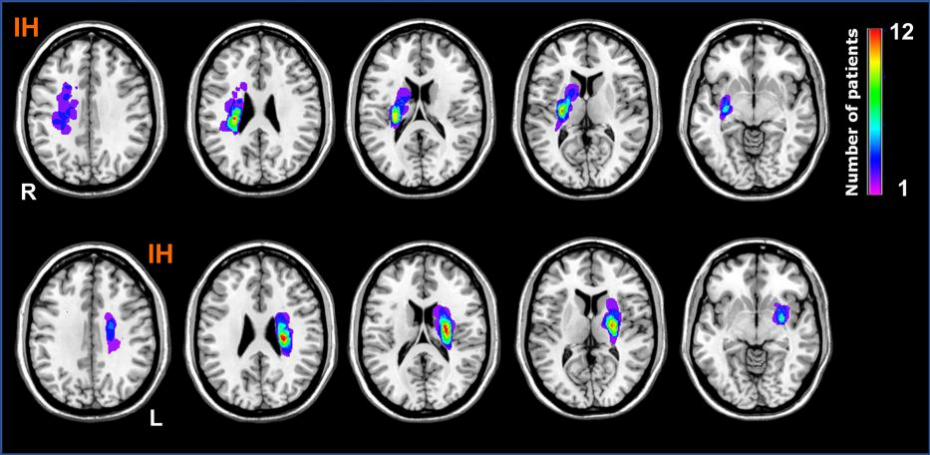
**Stroke lesion map. Heatmap of infarct lesions in people with stroke with right (*n* = 18) and left (*n* = 19) hemisphere infarction overlaid on a standardized template (MRIcron®).** All locations were subcortical regions in the middle cerebral artery territory, mostly involving the corona radiata and basal ganglia. IH, ipsilesional hemisphere; L, left; R, right.

A total of 37 (25 male) people with stroke (18 right hemispheric strokes) and 22 age- and sex-matched controls were enrolled (Table 1). People with stroke had a median age of 60 years (range 50–66), a median of 22 days (17–25) after stroke onset, a median National Institutes of Health Stroke Scale (NIHSS) of 4 (2–7), an ARAT of 32 (6–52) and a FM-UE of 50 (23–62) at enrolment. All people with stroke had standard clinical care, which consists of 60–120 min of therapy at least 3 days per week for up to 6 months after stroke. People with stroke showed statistically significant improvements in both ARAT and FM-UE scores between 3 and 12 weeks after stroke (*Z* = 4.8 and 5.2 respectively, both *P* < 0.001). Fourteen of 29 people with stroke showed clinically significant improvements (12 points for the dominant and 17 points for non-dominant side^32^) in ARAT score, and 14/29 people with stroke showed clinically significant improvements (>12.4 points^33^) in FM-UE. Two patients showed a clinically significant improvement in the ARAT but not FM-UE and two in the FM-UE but not the ARAT. No significant difference in motor scores was noted between people with stroke with right or left hemispheric strokes (all *P* > 0.5). The group lesion map (Fig. 1) showed a homogenous grouping, with infarctions in the corona radiata and basal ganglia within the MCA territory without cortical involvement. All 37 people with stroke had resting MEG recordings in Week 3, with 16 people with stroke having also resting MEG recordings at Week 12. Participants declined the second scan for a variety of reasons, including transfer to other hospitals or to home. For stroke people with or without 12-week follow-up, the HMM metrics at post-stroke Week 3 showed no group difference (see Supplementary Table 1).

**Table 1:** Participant characteristics

Participant	Age (years)	Sex	Affected hand	Time post-stroke (days)	Day 0	Weeks 2∼4			Week 12	
					NIHSS	mRS	ARAT	FM-UE	ARAT	FM-UE
1	49	M	R	26	8	3	3	18	9	33
2^a^	50	M	R	24	5	3	17	38	41	51
3^a^	41	F	R	28	5	3	3	20	9	33
4	57	M	L	17	3	2	38	50		
5^a^	75	F	L	20	5	3	20	20	35	33
6	59	M	R	14	2	2	57	66		
7	61	M	R	25	1	1	57	66	57	66
8^a^	68	F	L	20	1	1	54	58	57	66
9^a^	55	F	L	24	6	3	13	50	55	65
10	52	M	L	14	3	3	34	52	53	64
11^a^	56	M	R	16	1	2	54	65	57	66
12	75	M	L	27	12	4	4	21		
13	74	F	L	23	7	4	6	23		
14^a^	61	M	R	28	4	3	3	16	20	50
15	34	M	R	21	4	1	57	65	57	66
16^a^	64	M	L	15	8	4	3	13	6	18
17	73	M	L	24	2	4	47	54		
18	41	M	R	27	11	4	9	18		
19	46	M	R	20	3	1	57	66		
20	67	M	R	28	3	4	56	63	57	66
21	68	F	L	14	2	4	57	62	57	66
22	41	F	R	25	15	5	24	49	38	62
23^a^	50	M	L	17	7	4	7	32	27	47
24	31	M	L	23	2	4	57	63	57	65
25^a^	63	M	R	24	7	4	4	12	22	39
26	66	M	L	20	7	4	3	24	38	59
27^a^	60	M	L	17	6	4	3	26	28	49
28	75	F	L	15	8	4	38	43	57	63
29	70	M	R	19	3	4	6	32	48	47
30	44	F	R	23	3	4	56	60	57	64
31	56	F	R	18	1	3	57	64	57	66
32^a^	63	M	R	28	2	3	32	53	52	57
33^a^	62	F	L	26	5	3	57	61	57	66
34^a^	59	M	R	20	3	4	3	8	8	18
35^a^	61	M	R	28	12	4	20	40	56	54
36	51	F	L	10	2	3	34	52		
37^a^	60	M	L	22	2	3	54	65	57	66
People with stroke (*n* = 37)	60 (50–66)	25 M/12 F	18 L/19 R	22 (17–25)	4 (2–7)	3 (3–4)	32 (6–52)	50 (23–62)	52 (25–57)	59 (45–66)
Control (*n* = 22)	61 (52–62)	12 M/10 F								

Demographics for people with stroke. Data are presented as the median with interquartile range.

ARAT, Action Research Arm Test; F, female; FM-UE, Fugl-Meyer upper extremity score; L, left; M, male; mRS, modified Rankin Scale; NIHSS, National Institutes of Health Stroke Scale; R, right.

^a^Subjects with two (3 weeks and 12 weeks post-stroke) resting-state magnetoencephalography evaluations.

### HMM identified resting-state SMNs

**Figure 2:**
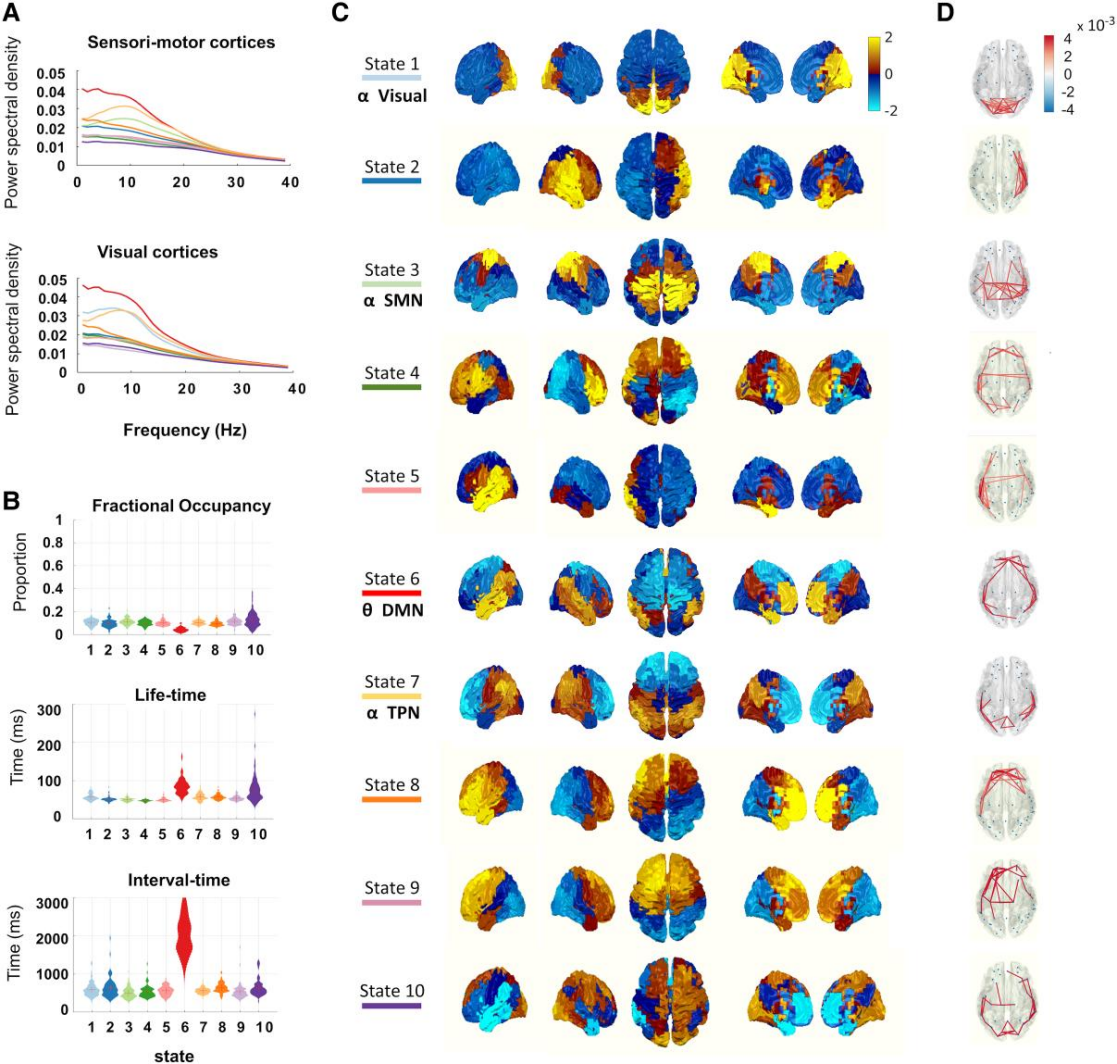
**HMM-derived MEG resting-state networks.** Resting-state MEG signals concatenated from all participants including controls (*n* = 22) and people with stroke (*n* = 37 at Week 3, *n* = 16 at Week 12) characterized by 10 HMM states. (**A**) Spectral profiles, (**B**) temporal metrics and (**C**–**D**) spatial profiles. The major resting-state networks include an SMN (State 3), a temporo-parietal network (TPN, State 7), a VN (state 1) and a DMN (State 6). Their spatial activation profiles resemble those of resting-state fMRI networks.^35^.

First, to determine whether we could identify a SMN in our resting MEG data, all resting-state data (i.e. two timepoints for the people with stroke and one timepoint for the controls) were submitted to a time delay embedded HMM. Ten resting-state networks were decomposed with corresponding spectral profiles (Fig. 2A), spatial profiles (Fig. 2C-D) and temporal metrics (Fig. 2B), which replicated the pattern showed by Vidaurre *et al*.^34^ before. We identified a resting-state SMN, with a frequency peak (10 Hz) in the α- (μ-) frequency range (State 3). The spatial distribution of this α-SMN is compatible with that seen in a previous resting-state fMRI study.^35^ This initial proof-of-principle analysis demonstrated that SMNs could be identified in our data.

**Figure 3:**
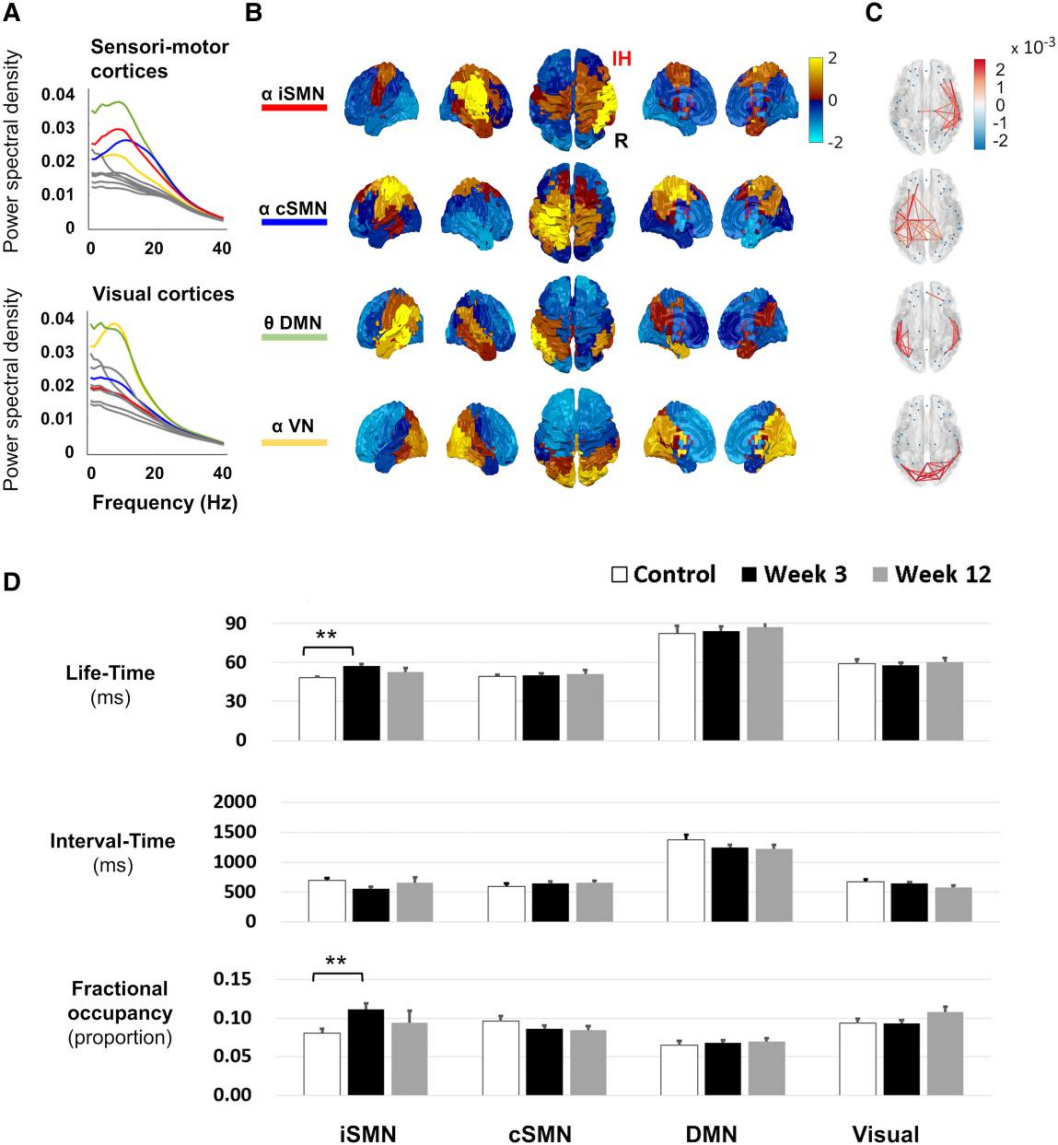
**MEG resting-state SMNs from people with right hemispheric strokes.** The major resting-state networks from people with stroke with right MCA infarction (*n* = 18 at Week 3, *n* = 8 at Week 12) and controls (*n* = 22), with corresponding (**A**) spectral profiles, (**B**) spatial profiles, (**C**) spatial coherence and (**D**) temporal metrics. Shown are the iSMN, cSMN, DMN and VN. Full 2 × 2 mixed design ANOVAs were run for each temporal metric (life-time, interval time, FO) separately, with a between-subject factor of group (controls, either 3 or 12 weeks post-stroke) and a within-subject factor of regions (iSMN, cSMN). For full statistical results, see Supplementary Table 2. Bonferroni-corrected *post hoc* tests demonstrated significantly increased life-time and FO in iSMN at 3 weeks post-stroke when compared with controls (life-time: U = 345, *P* < 0.001, *r* = 0.63; FO: U = 320, *P* < 0.001, *r* = 0.52). IH, ipsilesional hemisphere; L, left; R, right. *P < 0.05, **P < 0.01.

**Figure 4:**
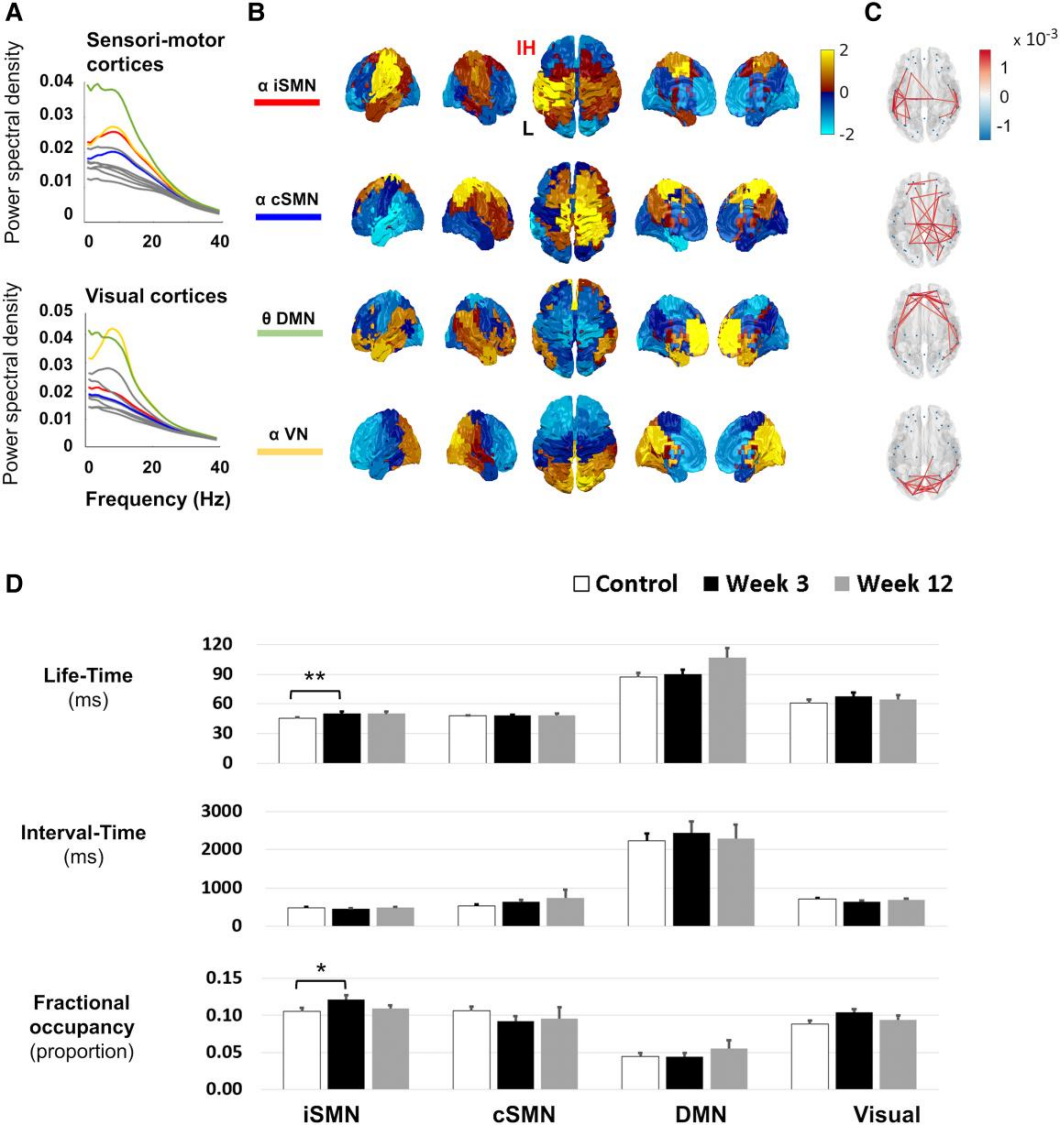
**MEG resting-state SMNs from people with left hemispheric strokes.** The major resting-state networks from people with stroke with left MCA infarction (*n* = 19 at Week 3, *n* = 8 at Week 12) and controls (*n* = 22), with corresponding (**A**) spectral profiles, (**B**) spatial profiles, (**C**) spatial coherence and (**D**) temporal metrics. Shown are the iSMN, cSMN, DMN and VN. Full 2 × 2 mixed design ANOVAs were run for each temporal metric (life-time, interval time, FO) separately, with a between-subject factor of group (controls, either 3 or 12 weeks post-stroke) and a within-subject factor of regions (iSMN, cSMN). For full statistical results, see Supplementary Table 2. Bonferroni-corrected *post hoc* tests demonstrated significantly increased life-time and FO in iSMN. Significantly increased life-time and FO were noted in iSMN at 3 weeks post-stroke when compared with controls (life-time: U = 105, *P* = 0.007, *r* = 0.42; FO: U = 288, *P* = 0.039, *r* = 0.32). IH, ipsilesional hemisphere; L, left; R, right. *P < 0.05, **P < 0.01.

Our cohort contained people with both left and right hemisphere strokes. We reasoned that ipsilesional and contralesional hemispheres might be expected to show distinct changes post-stroke.^12,36,37^ Therefore, we next applied two separate time delay embedded HMM analysis for people with stroke with right (*n* = 18; Fig. 3) and left (*n* = 19; Fig. 4) MCA infarctions, both concatenated with control data (*n* = 22). For both groups, it was possible to identify an iSMN and a contralesional SMN (cSMN), in the α-frequency range, which had characteristic spectral and spatial features (Figs. 3A–C and 4A–C).

To test for specificity of any results involving the SMNs, we defined two control states, i.e. two robust RSNs, that did not include motor areas. We found a clear visual network (VN) in the α-frequency range (10 Hz) (Figs 2–4) comprising primary and associated visual cortices. Further, we identified a default mode network (DMN) in the θ frequency range (3–5 Hz) (Figs 2–4), which also shared common spatial distribution as seen in resting-state fMRI^35^ and was characterized by the longest life-time and interval time among all HMM states.

### Post-stroke life-time and FO of ipsilesional resting-state SMNs are increased compared with controls and contralateral resting-state networks

Having identified robust SMNs, we next wished to investigate whether they changed after stroke. To this end, we extracted HMM metrics (life-time, interval time and FO) from the iSMN and cSMN separately for each subject and timepoint. We then pooled together the data from right (*n* = 18) and left (*n* = 19) hemispheric people with stroke.

We ran three 2 × 2 mixed design ANOVAs, one for each temporal metric (life-time, interval time, FO), with a between-subject factor of group (controls, people with stroke at 3 weeks post-stroke) and a within-subject factor of regions (iSMN, cSMN). We found a significant main effect for group in life-time (*F*(1,79) = 16.27, *P* < 0.001, η_p_^2^ = 0.171) but not for FO or interval time (*P*’s > 0.5; see Supplementary Table 2 for full ANOVA statistics), indicating longer state visits in people with stroke. Further, we found differences between iSMN and cSMN (life-time: *F*(1,79) = 3.76, *P* = 0.056, η_p_^2^ = 0.045, longer life-times for iSMN; FO: *F*(1,79) = 6.55, *P* = 0.012, η_p_^2^ = 0.077, higher FO for iSMN; interval time: *F*(1,79) = 6.47, *P* = 0.013, η_p_^2^ = 0.076, longer interval time for cSMN). Moreover, there were significant region × group interactions in life-time (*F*(1,79) = 16.4, *P* < 0.001, η_p_^2^ = 0.172), FO (*F*(1,79) = 20.77, *P* < 0.001, η_p_^2^ = 0.208) and interval time (*F*(1,79) = 13.1, *P* < 0.001, η_p_^2^ = 0.142). Interactions were followed up using non-parametric Mann–Whitney U tests (corrected α = 0.025). Life-time and FO was significantly greater people with stroke than controls in iSMN (U = 342, *P* < 0.001, *r* = 0.497; U = 449, *P* < 0.001, *r* = 0.385, respectively) but comparable in cSMN (U = 771, *P* = 0.684, *r* = 0.045; U = 655, *P* = 0.131, *r* = 0.168, respectively). The interval time showed no group difference in either iSMN (U = 628, *P* = 0.078, *r* = 0.196) or cSMN (U = 1030, *P* = 0.041, *r* = 0.228). For completeness, the results for the same analysis at 12 weeks post-stroke is reported in the Supplementary material which showed the life-time was still significantly greater in people with stroke than controls in iSMN (U = 210, *P* = 0.018, *r* = 0.31) but not in cSMN (U = 326, *P* = 0.66, *r* = 0.06).

The same analysis applied in the control states (DMN, VN) showed no significant main effects of group or region × group interactions (all *P*’s > 0.1) in either of three HMM metrics. However, significant main effects of region between DMN and VN were noted in all temporal metrics (all *P*’s < 0.001; see Supplementary Table 2 for full statistics), indicating longer life-time, longer interval time and lower FO in DMN, compared with VN. Having established the importance of the ipsilateral SMN, we next evaluated its temporal properties in more detail.

### Life-times of iSMN depend on lesion side

To determine whether the post-stroke change of life-time and FO observed in iSMN was dependent on the side of the lesion, we ran Mann–Whitney U tests. The life-time and FO of iSMN was significantly greater in people with stroke with either right MCA infarction (U = 345, *P* < 0.001, *r* = 0.63; U = 320, *P* < 0.001, *r* = 0.52, respectively) (Fig. 3D) or left MCA infarction (U = 105, *P* = 0.007, *r* = 0.42; U = 288, *P* = 0.039, *r* = 0.32, respectively) than controls (Fig. 4D). We further explored if there were differences between right and left MCA infarctions in life-time and FO. The Mann–Whitney U Tests showed that the life-time was even more increased in left than right MCA infarction (U = 73, *P* = 0.003, *r* = 0.49), but the FO showed no side difference (U = 221, *P* = 0.129, *r* = 0.25).

### iSMN life-time and FO trend towards normalizing during stroke recovery

We next went on to investigate how the resting-state networks change during stroke recovery. To this end, we performed Wilcoxon signed rank test for temporal metrics (life-time, interval time, FO) separately. The life-time and FO of iSMN significantly decreased at post-stroke Week 12 when compared with Week 3 (*Z* = 2.07, *P* = 0.038, *r* = 0.52; *Z* = 2.23, *P* = 0.026, *r* = 0.56, respectively), with no difference in interval time (*Z* = 1.34, *P* = 0.18, *r* = 0.34).

### iSMN life-time and FO correlate with function in people with stroke with not fully recovered hand paresis

**Figure 5:**
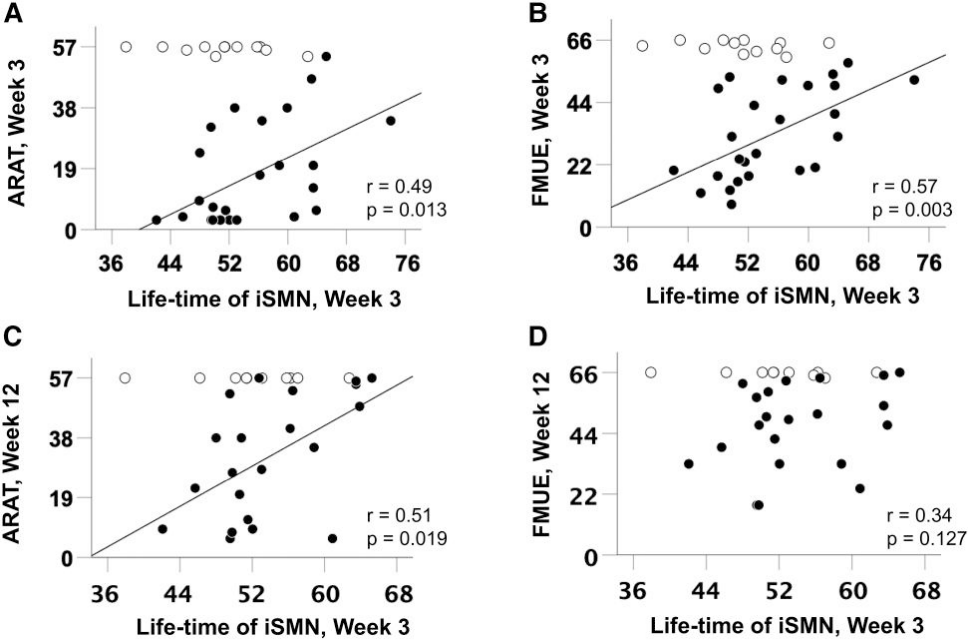
**Correlation between functional scores and ipsilesional resting-state SMNs.** The mean life-time of iSMN at Week 3 after stroke in people with stroke showed a significantly positive correlation with concurrent (**A**) ARAT score and (**B**) FM-UE score, in those with initial moderate to severe hand paresis (*n* = 25, dark circle ●) as defined by ARAT score < 56 and FM-UE score < 65 at stroke Week 3. Such functional correlations were not seen in stroke survivors with initial nearly fully recovered hand paresis (*n* = 12, white circle ○), defined by ARAT score of 56–57 or FM-UE score of 65–66 at stroke Week 3. (**C**) The life-time of iSMN at stroke Week 3 also positively correlated with ARAT at stroke Week 12 in those with initial moderate to severe hand paresis (*n* = 21, dark circle ●) but not (**D**) FM-UE at stroke Week 12. The FO of iSMN showed similar results as the life-time (see Supplementary Fig. 1).

Given the specific finding that the life-time and FO of iSMN was changed early post-stroke, we next investigated whether life-time of iSMN relates to clinical recovery. Clinical recovery was assessed by two behavioural measures: the FM-UE and the ARAT. As we hypothesized that people with stroke with very good recovery might be at ceiling on our scores, we divided our cohort of people with stroke into two groups: those with an initially nearly fully recovered hand paresis, defined as ARAT score > 55 or FM-UE score > 64 at stroke Week 3 (*n* = 12, open circles), and those with not fully recovered hand paresis, defined as ARAT score < 56 and FM-UE score < 65 (*n* = 25, closed circles) in Fig. 5.

In people with stroke with initially not fully recovered hand paresis, life-time and FO of iSMN at Week 3 were positively correlated with ARAT at Week 3 (life-time: *r* = 0.49, *P* = 0.013, Fig. 5A; FO: *r* = 0.43, *P* = 0.030, Supplementary Fig. 1A) and FM-UE at Week 3 (life-time: *r* = 0.57, *P* = 0.003, Fig. 5B; FO: *r* = 0.44, *P* = 0.029, Supplementary Fig. 1B). Further correlation analysis was used to determine if early resting-state network related to final motor outcome, as a biomarker of stroke recovery. Week 3 iSMN life-time and FO were significantly positively correlated with Week 12 ARAT in people with stroke with initially not fully recovered hand paresis (life-time: *r* = 0.51, *P* = 0.019, Fig. 5C; FO: *r* = 0.46, *P* = 0.036, Supplementary Fig. 1C) but not with FM-UE (life-time: *r* = 0.34, *P* = 0.127, Fig. 5D; FO: *r* = 0.40, *P* = 0.071, Supplementary Fig. 1D). We then wished to know whether Week 3 iSMN life-time and FO could independently predict behavioural recovery. We performed a partial correlation analysis between iSMN life-time and FO at Week 3 and ARAT at Week 12, controlling for ARAT at Week 3, and demonstrated that the relationship was no longer significant (life-time: *r* = 0.42, *P* = 0.063; FO: *r* = 0.38, *P* = 0.096).

### HMM resting-state iSMNs in motor task

Finally, while the strength of this study is that these metrics can be obtained at rest, making them clinically feasible, we wanted to determine the face validity of our metrics, by investigating how these resting-state networks interacted during motor task as showed in Supplementary results.

## Discussion

This study characterized temporal features of post-stroke SMNs from resting-state brain activity. Robust resting-state networks with specific spatial, frequency and temporal features were extracted from whole brain inference of HMM. The life-time and FO of the resting-state iSMN was significantly increased at the subacute time period after stroke and correlated with motor functions specifically in people with stroke with moderate to severe hand paresis.

### Functionally correlated resting-state iSMN at subacute stroke

The resting-state SMN constructed from fMRI, EEG or MEG shares the same spatial distribution across the different modalities.^38,39^ In this study, the SMN arising from our HMM analysis (Fig. 2) could further be decomposed into ipsilesional and contralesional SMNs in people with stroke (Fig. 3 and 4); the two networks showed differential changes during recovery. Both the life-time and FO of the ipsilesional resting-state SMN were increased at 3 weeks after stroke as compared with controls. The increased recruitment of the iSMN was positively correlated with motor scores in people with subacute stroke with not fully recovered hand paresis and gradually decreased during stroke recovery. This pattern of changes would support the hypothesis that the increased recruitment of the iSMN in subacute stroke may reflect mechanisms of recovery after stroke. The finding of increased iSMN recruitment post-stroke which then reduces with recovery is in line with previous task fMRI^37,40^ and task MEG^28^ studies, which have showed greater activity in these networks during movement of the paretic hand early after stroke, which gradually decreases during subsequent recovery.

The life-time and FO of iSMN at Week 3 after stroke was also correlated with the motor outcome at Week 12 after stroke, suggesting that dynamics within the iSMN are not only relevant to current behaviour but relate to subsequent improvements. Although the correlation was no longer significant when controlling motor scores at Week 3 after stroke, this does not rule out the hypothesis that the iSMN dynamics are a marker of pro-plastic processes: if they indeed are mechanistically involved in functional recovery, then they would be expected to be tightly correlated with motor performance, as seen here. However, this hypothesis remains to be tested.

Several studies have demonstrated that early motor scores can independently predict motor outcome except in those people with severe limb paresis.^4,5,41^ The outliers in the proportional recovery model usually have FM-UE between 2 and 6,^4^ which is far more severe than our cohort. In this study, the life-time of resting-state iSMN correlated better in those with initially marked hand paresis (Fig. 5C) and could serve as a biomarker for those outliers in the proportional recovery model. People with stroke with more severe hand paresis should be enrolled in further study to verify this finding.

### Changes in network metrics were specific to the iSMN

In addition to the ipsilesional and contralesional SMNs, the most prominent and robust resting-state networks from our data were DMN and visual network. However, only the iSMN showed significant post-stroke changes and behaviour correlations in this study. The consistency of DMN and VN metrics between people with stroke and controls demonstrate the specificity of our findings in this population of people with motor deficits post-stroke and suggest that these changes were not driven by global differences between the group such as medications or drowsiness. However, DMN or VN may have post-stroke changes in different stroke cohorts with homogenous dysfunction related to DMN or VN.^17^

### Alterations in SMN dynamics may reflect disinhibition in subacute stroke

Previous resting-state TMS studies have demonstrated decreased ipsilesional MEP amplitude^5,42^ but also decreased intracortical and interhemispheric inhibition^43^ during the subacute recovery phase after stroke. Resting-state fMRI studies have shown decreased functional connectivity within the SMN, particularly between the hemispheres.^44^ Taken together, these findings highlight the disruption of functional networks, particularly a dissociation of the ipsilesional and contralesional motor cortices, accompanied with disinhibition phenomenon in the critical recovery period.^45^ The increased recruitment of iSMN observed here was particularly prominent early rather than later after stroke, especially in people with greater hand paresis. The relative decrease in the ability of the brain to switch between states and to remain locked in one state could plausibly be a reflection of a disinhibited cortex, where increased cortical activity leads to an amplification and perseverance of ongoing activity. It is possible, therefore, that the increased iSMN observed here reflects local disinhibition, potentially a reflection of cortical neuroplastic phenomena. The finding that the changes in iSMN life-time are greater in people with dominant rather than non-dominant hemisphere strokes would fit with this hypothesis, as the dominant hemisphere receives relatively less interhemispheric inhibition from the non-dominant hemisphere than vice versa. These results therefore add weight to the hypothesis that disinhibition leads to a dysregulation of sensorimotor connectivity, particularly reflected in decreased interhemispheric connections and increased local connectivity.

### HMM dynamics during motor task

**Figure 6:**
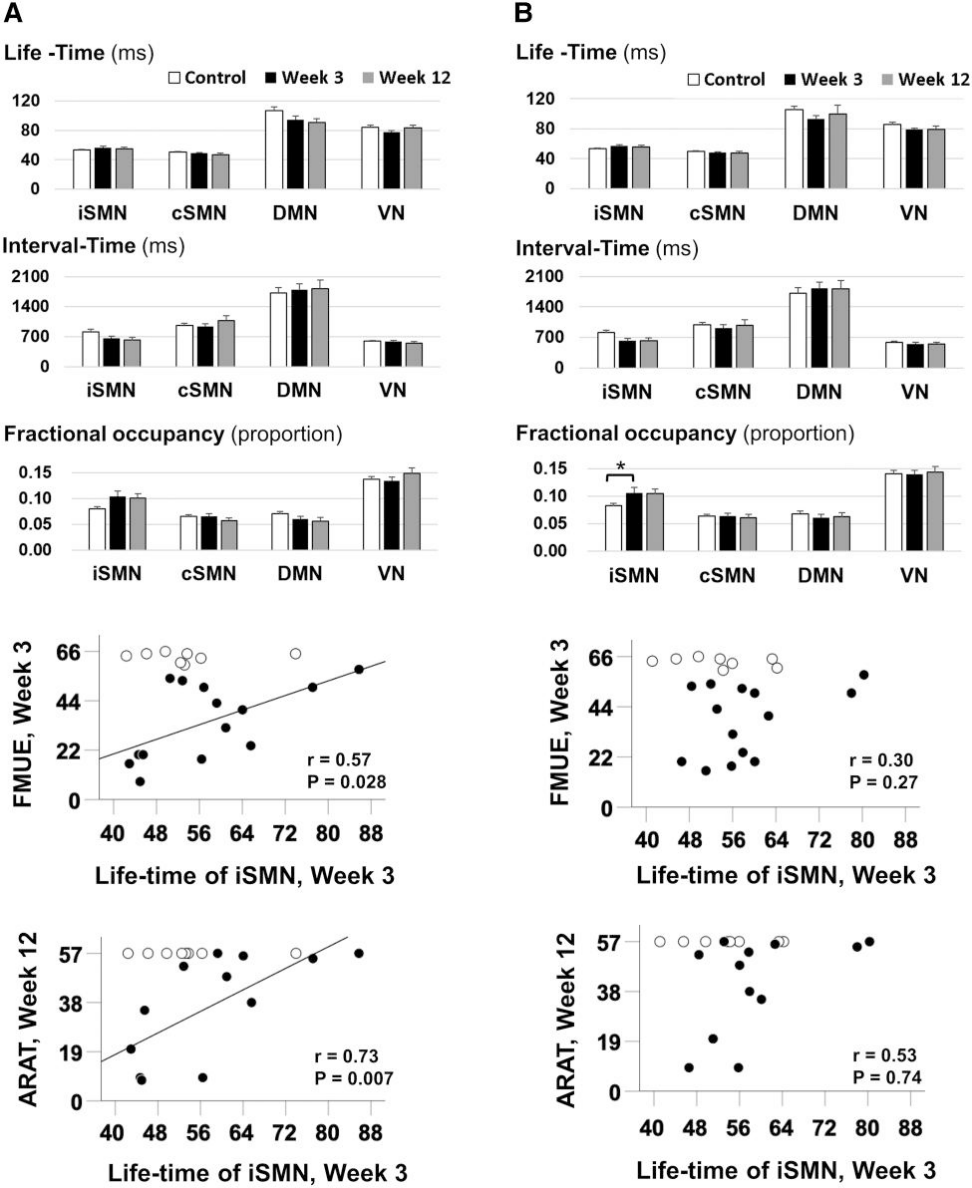
**The resting-state networks during motor task.** The decomposed HMM resting-state networks applied in participants during (**A**) paretic (or left in controls) hand movement task and (**B**) non-paretic (or right in controls) hand movement task disclosed similar but less significant changes than in the resting-state data of life-time and FO in iSMN. Full 2 × 2 mixed design ANOVAs were run for each temporal metric (life-time, interval time, FO) separately, with a between-subject factor of group (controls, either 3 or 12 weeks post-stroke) and a within-subject factor of regions (iSMN, cSMN). For full statistical results, see Supplementary information. There were no significant *post hoc* differences between the two groups in any HMM metric during movement of the paretic hand (**A**). Bonferroni-corrected *post hoc* tests demonstrated that during movement of the non-paretic hand, there was a significantly higher FO in iSMN in people with stroke at 3 weeks compared with controls (U = 586, *P* = 0.015, *r* = 0.32; **B**). The life-time of iSMN during paretic hand movement showed significant functional correlations, which were not seen during movement of the non-paretic hand. ARAT, Action Research Arm Test; cSMN, contralesional sensorimotor network; DMN, default mode network; FM-UE, Fugl-Meyer upper extremity; VN, visual network. *P < 0.05, **P < 0.01.

Neural activity acquired during task has given us rich information about recovery post-stroke. However, it is often difficult to acquire in the subacute phase and relies on people with stroke having sufficient function and cognitive ability to perform a task. In this study, therefore, we wanted to investigate whether resting data might be similarly informative. The HMM brain states decomposed from resting-state data were also applied in motor task data. We disclosed similar trend but less significant changes in iSMN after stroke. Importantly, the life-time of resting-state iSMN applied during task also correlated with motor scores but only during paretic hand movement rather than non-paretic hand movement (Fig. 6). These results suggest that the increased recruitment of SMN at rest may also be linked to paretic hand movement. However, further delicate state-shift studies are required to determine the relationship between resting-state SMN (α-range) and task-state SMN (β-range).

### Considerations and further applications

The people with stroke in this study mostly had mild or moderate hand paresis, with only few with FM-UE < 11.^46^ This relatively well-recovered group may lead us to underestimate the correlation power of the life-time or FO. In addition, the assumptions in the HMM may limit the interpretation of results in a number of ways. Firstly, a known limitation of HMM inference is the *a priori* selection of the number of HMM states. Here, we inferred 10 HMM states as 10 HMM states yielded 1 ipsilesional and 1 contralateral SMN. Previous studies have validated the robustness of results using a range of HMM states and found that varying the number of states (within a reasonable range, i.e. 3–14) did not change the results.^23^ Secondly, HMM assumes that the states are mutually exclusive and only a single state is active at a single timepoint, which excludes the possibility that multiple networks may be active at the same time, which may be an over-simplification. New methods being developed should overcome this limitation and are likely to be important in future studies.^47^ Finally, our cohort only included people with subcortical strokes. It is not clear how these findings would translate to people with cortical lesions, although given that the HMM approach is data-driven and does not rely on ROIs or assumptions about anatomy, it is likely that similar changes in dynamics would be observed.

## Conclusion

We have demonstrated changes in the dynamics and connectivity of the iSMN early after stroke at rest. The increased life-time and FO of resting-state iSMN positively correlated with motor functions in people with stroke with moderate hand paresis, suggesting that network dynamics may have a functional relevance. Our findings are in line with those from other modalities, suggesting that local disinhibition and interhemispheric dysconnectivity are prominent after stroke and relate to motor function. That we can identify these markers in resting electrophysiological data overcomes many of the issues associated with task performance and changes in neurovascular coupling in people with stroke and therefore offers substantial promise to study recovery after stroke.

## Supplementary Material

fcae011_Supplementary_Data

## Data Availability

We will consider requests to access the raw data in a trusted research environment as part of a collaboration. Please contact I.-H.L. with all requests.
