# Supplementary material for: Post-stroke upper limb recovery is correlated with dynamic resting-state network connectivity
Source: Brain Commun. 2024 Jan 23;6(1):fcae011. doi: 10.1093/braincomms/fcae011 (PMC10853981; doi:10.1093/braincomms/fcae011)
Supplement: fcae011_Supplementary_Data [file fcae011_supplementary_data.docx]

# Supplementary Materials

## Analysis of resting-state data comparisons between controls and 12 week post-stroke

We first ran three 2x2 mixed-design ANOVAs one for each temporal metric (life-time, interval-time, fractional occupancy), with a between-subject factor of group (controls, 12 week post-stroke) and a within-subject factor of regions (iSMN, cSMN). There was a significant main effect for group in life-time (F(1,58)=5.76, p=0.02, η_p_^2^=0.09, longer life-times in people with stroke), but not in fractional occupancy (F(1,58)=0.057, p=0.812, η_p_^2^=0.001) or interval-time (F(1,58)=1.23, p=0.272, η_p_^2^=0.021). A significant main effect of region indicates longer life-times, longer interval-times, and lower fractional occupancy and in DMN, compared to VN (all p’s <0.001). There were no significant region x group interactions in life-time, fractional occupancy and interval-time (F(1,58)=2.18, p=0.145, η_p_^2^=0.036; F(1,58)=3.97, p=0.051, η_p_^2^=0.064; F(1,58)=4.03, p=0.050, η_p_^2^=0.065). Further testing (corrected α=0.025) revealed that the life-time was significantly greater in people with stroke than controls in iSMN (U=210, p=0.018, r=0.31), but not in cSMN (U=326, p=0.66, r=0.06). The same analysis applied in controls regions (DMN, VN) showed no significant region x group interactions or main effect in group in any HMM metric (all p’s > 0.1).

## HMM resting-state ipsilesional sensorimotor networks in motor task

Finally, to investigate whether the results generalise to data acquired during motor activation the HMM model inferred from resting state data was applied to motor task data. 20 controls and 28 people with stroke (12 right and 16 left MCA infarction) had MEG recordings of motor task after resting-state recording. First, we wished to investigate whether, as in our resting data, any of the temporal HMM metrics differed after stroke. We extracted each of our HMM metrics (life-time, interval-time and fractional occupancy) from the iSMN and cSMN separately for each subject. We then pooled together the data from right (n=12) and left (n=16) hemispheric stroke survivors. We therefore ran three 2x2 mixed design ANOVAs for temporal metrics (life-time, interval-time, fractional occupancy) separately, with a between-subject factor of group (controls, 3 week post-stroke) and a within-subject factor of regions (iSMN, cSMN). During paretic (or left in controls) hand movement, there was a significant main effect for group in fractional occupancy (F(1,63)=4.087, p=0.047, η_p_^2^=0.061), but not for life-time or interval-time (p’s>0.1, see Supplementary Table 2 for full ANOVA statistics), indicating higher fractional occupancy in people with stroke. Further, we found differences between iSMN and cSMN (life-time: F(1,63)=24.26, p<0.001, η_p_^2^=0.278, longer life-times for iSMN; fractional occupancy: F(1,63)=29.4, p<0.001, η_p_^2^=0.318, higher fractional occupancy for iSMN; interval-time: F(1,63)=18.81, p<0.001, η_p_^2^=0.23, longer interval-time for cSMN). Moreover, there were significant group x region interaction in fractional occupancy (F (1,63)=4.952, p=0.03, η_p_^2^=0.073), but not in life-time (F (1,63)=3.92, p=0.052, η_p_^2^=0.059) or interval-time (F(1,63)=1.63, p=0.21, η_p_^2^=0.025). Interaction was followed up using non-parametric Mann-Whitney U tests (corrected α=0.025). The fractional occupancy showed no group difference in either iSMN (U=685, p=0.027, r=0.27) or cSMN (U=524, p=0.94, r=0.01).

During nonparetic (or right in controls) hand movement, there was a significant main effect for group in fractional occupancy (F(1,58)=4.95, p=0.03, η_p_^2^=0.079) and interval-time (F(1,58)=4.17, p=0.046, η_p_^2^=0.067), but not for life-time (p’s>0.5, see Supplementary Table 2 for full ANOVA statistics), indicating higher fractional occupancy and shorter interval-time in patients. Further, we found differences between iSMN and cSMN (life-time: F(1,58)=41.35, p<0.001, η_p_^2^=0.416, longer life-times for iSMN; fractional occupancy: F(1,58)=46.72, p<0.001, η_p_^2^=0.446, higher fractional occupancy for iSMN; interval-time: F(1,58)=17.42, p<0.001, η_p_^2^=0.231, longer interval-time for cSMN). Moreover, there were significant group x region interaction in fractional occupancy, which was followed up using non-parametric Mann-Whitney U tests (corrected α=0.025). The fractional occupancy showed a significant group difference in iSMN fractional occupancy [iSMN: U=586, p=0.015, r=0.32; cSMN: U=434, p=0.90, r=0.017], indicated higher fractional occupancy in patients.

The same analysis applied in controls networks (DMN, VN) showed no significant effect of group, or region x group interactions on either of the HMM metrics (all p’s > 0.09), but a significant main effect of region, indicating longer life-times, longer interval-times and lower fractional occupancy in DMN, compared to VN (all p’s <0.001) during either hand movement (Fig 6A-B).

We then performed a similar analysis at week 12. There were no significant differences between controls and people with stroke at week 12 among life-time, fractional occupancy or interval-time in sensorimotor regions (iSMN, cSMN) or control regions (DMN, VN), with all testing p’s > 0.05. To evaluate the changes of metrics during recovery, we performed Wilcoxon Signed Rank Test for temporal metrics (life-time, interval-time, fractional occupancy) separately. All the temporal metrics showed no significant changes in iSMN or cSMN (all p’s >0.5).

We then went on to investigate the relationship between these HMM metrics and clinical scores. Similarly, to the resting state data, the iSMN life-time during paretic hand movement at week 3 was positively correlated with FM-UE at week 3 (r=0.57, p=0.028, Fig. 6A) in moderate-to-severe people with stroke. However, there was no correlation between the iSMN life-time during paretic hand movement and ARAT at week 3 (r=0.38, p=0.17). The iSMN life-time during paretic hand movement at stroke week 3 was also positively correlated with FMUE at week 12 in moderate-to-severe people with stroke (r=0.75, p=0.005) and ARAT at week 12 (0.73, p=0.007). However, the correlations between iSMN and FM-UE or ARAT at week 12 were no longer significant when week 3 ARAT or FM-UE were included in the model. No correlations with clinical scores were noted in fractional occupancy or interval-time during either hand movements or life-time during non-paretic hand movements (Fig. 6B).

## Supplementary Table 1. Comparison of HMM metrics for people with stroke who are not fully recovered, with and without 12 week follow-up

|  | IH-LF | CH-LF | IH-FO | CH-FO | IH-IT | CH-IT |
| --- | --- | --- | --- | --- | --- | --- |
| 3 weeks after stroke **(N=25)** | **53.845**  ± 1.223 | **49.982**  ± 0.823 | **0.117**  ± 0.005 | **0.089**  ± 0.004 | **498.359**  ± 20.401 | **640.783**  ± 35.107 |
| 3 weeks after stroke **(N=16)*** | **53.845**  ± 1.745 | **50.133**  ± 1.595 | **0.114**  ± 0.008 | **0.093**  ± 0.006 | **518.098**  ± 36.984 | **633.198**  ± 60.189 |
| 12 weeks after stroke (N=16)***** | 51.230  ± 1.814 | 49.950  ± 1.697 | 0.102  ± 0.008 | 0.089  ± 0.009 | 571.135  ± 48.214 | 699.255  ± 99.223 |
| Control (N=22) | 46.842  ± 0.754 | 48.555  ± 0.658 | 0.093  ± 0.008 | 0.101  ± 0.009 | 585.174  ± 29.681 | 560.337  ± 29.999 |

IH = ipsilesional hemisphere; CH = contralesional hemisphere; LF = life-time; FO = fractional occupancy; IT = interval time; *people with stroke who participated in scanning at both 3 weeks and 12 weeks.

## Supplementary Table 2. Full statistics of ANOVA

**Rest-state 2x2 ANOVA for Group and Region (iSMN, cSMN) at 3 weeks**

|  | df | Life-time | | |  | Fractional occupancy | | |  | Interval-time | | |
| --- | --- | --- | --- | --- | --- | --- | --- | --- | --- | --- | --- | --- |
|  |  | F | p | η_p_^2^ |  | F | p | η_p_^2^ |  | F | p | η_p_^2^ |
| Group | 1,79 | 16.27 | <0.001 | 0.171 |  | 1.28 | 0.262 | 0.016 |  | 0.008 | 0.928 | 0.000 |
| Region | 1,79 | 3.76 | 0.056 | 0.045 |  | 6.55 | 0.012 | 0.077 |  | 6.47 | 0.013 | 0.076 |
| Group x Region | 1,79 | 16.4 | <0.001 | 0.172 |  | 20.77 | <0.001 | 0.208 |  | 13.1 | 0.001 | 0.142 |

**Rest-state 2x2 ANOVA for Group and Region (DMN, VN) at 3 weeks**

|  | df | Life-time | | |  | Fractional occupancy | | |  | Interval-time | | |
| --- | --- | --- | --- | --- | --- | --- | --- | --- | --- | --- | --- | --- |
|  |  | F | p | η_p_^2^ |  | F | p | η_p_^2^ |  | F | p | η_p_^2^ |
| Group | 1,79 | 0.58 | 0.45 | 0.007 |  | 1.26 | 0.265 | 0.016 |  | <0.001 | 0.993 | 0.000 |
| Region | 1,79 | 143.24 | <0.001 | 0.645 |  | 120.78 | <0.001 | 0.605 |  | 118.92 | <0.001 | 0.601 |
| Group x Region | 1,79 | 0.036 | 0.851 | <0.001 |  | 1.11 | 0.295 | 0.014 |  | 0.221 | 0.64 | 0.003 |

**Rest-state 2x2 ANOVA for Group and Region (iSMN, cSMN) at 12 weeks**

|  | df | Life-time | | |  | Fractional occupancy | | |  | Interval-time | | |
| --- | --- | --- | --- | --- | --- | --- | --- | --- | --- | --- | --- | --- |
|  |  | F | p | η_p_^2^ |  | F | p | η_p_^2^ |  | F | p | η_p_^2^ |
| Group | 1,58 | 5.76 | 0.02 | 0.09 |  | 0.057 | 0.812 | 0.001 |  | 1.23 | 0.272 | 0.021 |
| Region | 1,58 | 0.046 | 0.831 | 0.001 |  | 0.246 | 0.622 | 0.004 |  | 1.84 | 0.181 | 0.031 |
| Group x Region | 1,58 | 2.18 | 0.145 | 0.036 |  | 3.973 | 0.051 | 0.064 |  | 4.03 | 0.050 | 0.065 |

**Rest-state 2x2 ANOVA for Group and Region (DMN, VN) at 12 weeks**

|  | df | Life-time | | |  | Fractional occupancy | | |  | Interval-time | | |
| --- | --- | --- | --- | --- | --- | --- | --- | --- | --- | --- | --- | --- |
|  |  | F | p | η_p_^2^ |  | F | p | η_p_^2^ |  | F | p | η_p_^2^ |
| Group | 1,58 | 1.93 | 0.170 | 0.032 |  | 2.48 | 0.121 | 0.041 |  | 0.239 | 0.627 | 0.004 |
| Region | 1,58 | 114.04 | <0.001 | 0.663 |  | 59.75 | <0.001 | 0.507 |  | 80.73 | <0.001 | 0.582 |
| Group x Region | 1,58 | 3.12 | 0.083 | 0.051 |  | 0.09 | 0.768 | 0.002 |  | 0.003 | 0.954 | <0.001 |

**Task-state, paretic hand movement, 2x2 ANOVA for Group and Region (iSMN, cSMN) at 3 weeks**

|  | df | Life-time | | |  | Fractional occupancy | | |  | Interval-time | | |
| --- | --- | --- | --- | --- | --- | --- | --- | --- | --- | --- | --- | --- |
|  |  | F | p | η_p_^2^ |  | F | p | η_p_^2^ |  | F | p | η_p_^2^ |
| Group | 1,63 | 0.164 | 0.686 | 0.003 |  | 4.087 | 0.047 | 0.061 |  | 2.212 | 0.142 | 0.034 |
| Region | 1,63 | 24.26 | <0.001 | 0.278 |  | 29.4 | <0.001 | 0.318 |  | 18.81 | <0.001 | 0.23 |
| Group x Region | 1,63 | 3.92 | 0.052 | 0.059 |  | 4.952 | 0.03 | 0.073 |  | 1.634 | 0.206 | 0.025 |

**Task-state, paretic hand movement, 2x2 ANOVA for Group and Region (DMN, VN) at 3 weeks**

|  | df | Life-time | | |  | Fractional occupancy | | |  | Interval-time | | |
| --- | --- | --- | --- | --- | --- | --- | --- | --- | --- | --- | --- | --- |
|  |  | F | p | η_p_^2^ |  | F | p | η_p_^2^ |  | F | p | η_p_^2^ |
| Group | 1,63 | 4.969 | 0.029* | 0.073 |  | 1.632 | 0.206 | 0.025 |  | 0.156 | 0.694 | 0.002 |
| Region | 1,63 | 25.061 | <0.001 | 0.285 |  | 155.74 | <0.001 | 0.712 |  | 165.83 | <0.001 | 0.725 |
| Group x Region | 1,63 | 0.444 | 0.508 | 0.007 |  | 0.403 | 0.528 | 0.006 |  | 0.361 | 0.55 | 0.006 |

* Post-hoc non-parametric Mann-Whitney U tests (corrected α=0.025) showed no group difference for either DMN

(U=392, p=0.095, r=0.207) or VN (U=412, p=0.16, r=0.174).

**Task-state, non-paretic hand movement, 2x2 ANOVA for Group and Region (iSMN, cSMN) at 3 weeks**

|  | df | Life-time | | |  | Fractional occupancy | | |  | Interval-time | | |
| --- | --- | --- | --- | --- | --- | --- | --- | --- | --- | --- | --- | --- |
|  |  | F | p | η_p_^2^ |  | F | p | η_p_^2^ |  | F | p | η_p_^2^ |
| Group | 1,58 | 0.44 | 0.512 | 0.007 |  | 4.95 | 0.03 | 0.079 |  | 4.17 | 0.046 | 0.067 |
| Region | 1,58 | 41.35 | <0.001 | 0.416 |  | 46.72 | <0.001 | 0.446 |  | 17.42 | <0.001 | 0.231 |
| Group x Region | 1,58 | 8.83 | 0.004 | 0.132 |  | 6.49 | 0.013 | 0.101 |  | 1.08 | 0.303 | 0.018 |

**Task-state, non-paretic hand movement, 2x2 ANOVA for Group and Region (DMN, VN) at 3 weeks**

|  | df | Life-time | | |  | Fractional occupancy | | |  | Interval-time | | |
| --- | --- | --- | --- | --- | --- | --- | --- | --- | --- | --- | --- | --- |
|  |  | F | p | η_p_^2^ |  | F | p | η_p_^2^ |  | F | p | η_p_^2^ |
| Group | 1,58 | 4.326 | 0.042* | 0.069 |  | 0.495 | 0.484 | 0.008 |  | 0.129 | 0.721 | 0.002 |
| Region | 1,58 | 19.866 | <0.001 | 0.255 |  | 158.94 | <0.001 | 0.733 |  | 137.45 | <0.001 | 0.703 |
| Group x Region | 1,58 | 0.533 | 0.468 | 0.009 |  | 0.245 | 0.623 | 0.004 |  | 0.525 | 0.472 | 0.009 |

* Post-hoc non-parametric Mann-Whitney U tests (corrected α=0.025) showed no group difference for either DMN

(U=321, p=0.112, r=0.205) or VN (U=355, p=0.284, r=0.138).

**Task-state, paretic hand movement, 2x2 ANOVA for Group and Region (iSMN, cSMN) at 12 weeks**

|  | df | Life-time | | |  | Fractional occupancy | | |  | Interval-time | | |
| --- | --- | --- | --- | --- | --- | --- | --- | --- | --- | --- | --- | --- |
|  |  | F | p | η_p_^2^ |  | F | p | η_p_^2^ |  | F | p | η_p_^2^ |
| Group | 1,46 | 0.084 | 0.774 | 0.002 |  | 0.808 | 0.373 | 0.017 |  | 0.216 | 0.645 | 0.005 |
| Region | 1.46 | 31.18 | <0.001 | 0.404 |  | 30.34 | <0.001 | 0.397 |  | 17.15 | <0.001 | 0.272 |
| Group x Region | 1,46 | 5.75 | 0.021 | 0.111 |  | 6.42 | 0.015 | 0.123 |  | 3.93 | 0.053 | 0.079 |

**Task-state, paretic hand movement, 2x2 ANOVA for Group and Region (DMN, VN) at 12 weeks**

|  | df | Life-time | | |  | Fractional occupancy | | |  | Interval-time | | |
| --- | --- | --- | --- | --- | --- | --- | --- | --- | --- | --- | --- | --- |
|  |  | F | p | η_p_^2^ |  | F | p | η_p_^2^ |  | F | p | η_p_^2^ |
| Group | 1,46 | 2.046 | 0.159 | 0.043 |  | 0.073 | 0.788 | 0.002 |  | 0.024 | 0.876 | 0.001 |
| Region | 1,46 | 7.994 | 0.007 | 0.148 |  | 89.535 | <0.001 | 0.661 |  | 87.057 | <0.001 | 0.654 |
| Group x Region | 1,46 | 1.821 | 0.184 | 0.038 |  | 1.806 | 0.186 | 0.038 |  | 0.276 | 0.602 | 0.006 |

**Task-state, non-paretic hand movement, 2x2 ANOVA for Group and Region (iSMN, cSMN) at 12 weeks**

|  | df | Life-time | | |  | Fractional occupancy | | |  | Interval-time | | |
| --- | --- | --- | --- | --- | --- | --- | --- | --- | --- | --- | --- | --- |
|  |  | F | p | η_p_^2^ |  | F | p | η_p_^2^ |  | F | p | η_p_^2^ |
| Group | 1,48 | 0.029 | 0.865 | 0.001 |  | 2.431 | 0.126 | 0.048 |  | 1.363 | 0.249 | 0.028 |
| Region | 1,48 | 22.902 | <0.001 | 0.323 |  | 31.664 | <0.001 | 0.397 |  | 13.589 | 0.001 | 0.221 |
| Group x Region | 1,48 | 4.115 | 0.048 | 0.079 |  | 4.803 | 0.033 | 0.091 |  | 1.448 | 0.235 | 0.029 |

**Task-state, non-paretic hand movement, 2x2 ANOVA for Group and Region (DMN, VN) at 12 weeks**

|  | df | Life-time | | |  | Fractional occupancy | | |  | Interval-time | | |
| --- | --- | --- | --- | --- | --- | --- | --- | --- | --- | --- | --- | --- |
|  |  | F | p | η_p_^2^ |  | F | p | η_p_^2^ |  | F | p | η_p_^2^ |
| Group | 1,48 | 0.794 | 0.377 | 0.016 |  | 0.065 | 0.80 | 0.001 |  | 0.067 | 0.797 | 0.001 |
| Region | 1,48 | 483.30 | 0.001 | 0.212 |  | 82.95 | <0.001 | 0.633 |  | 103.1 | <0.001 | 0.682 |
| Group x Region | 1,48 | 0.004 | 0.951 | <0.001 |  | 0.215 | 0.645 | 0.004 |  | 0.345 | 0.56 | 0.007 |

| 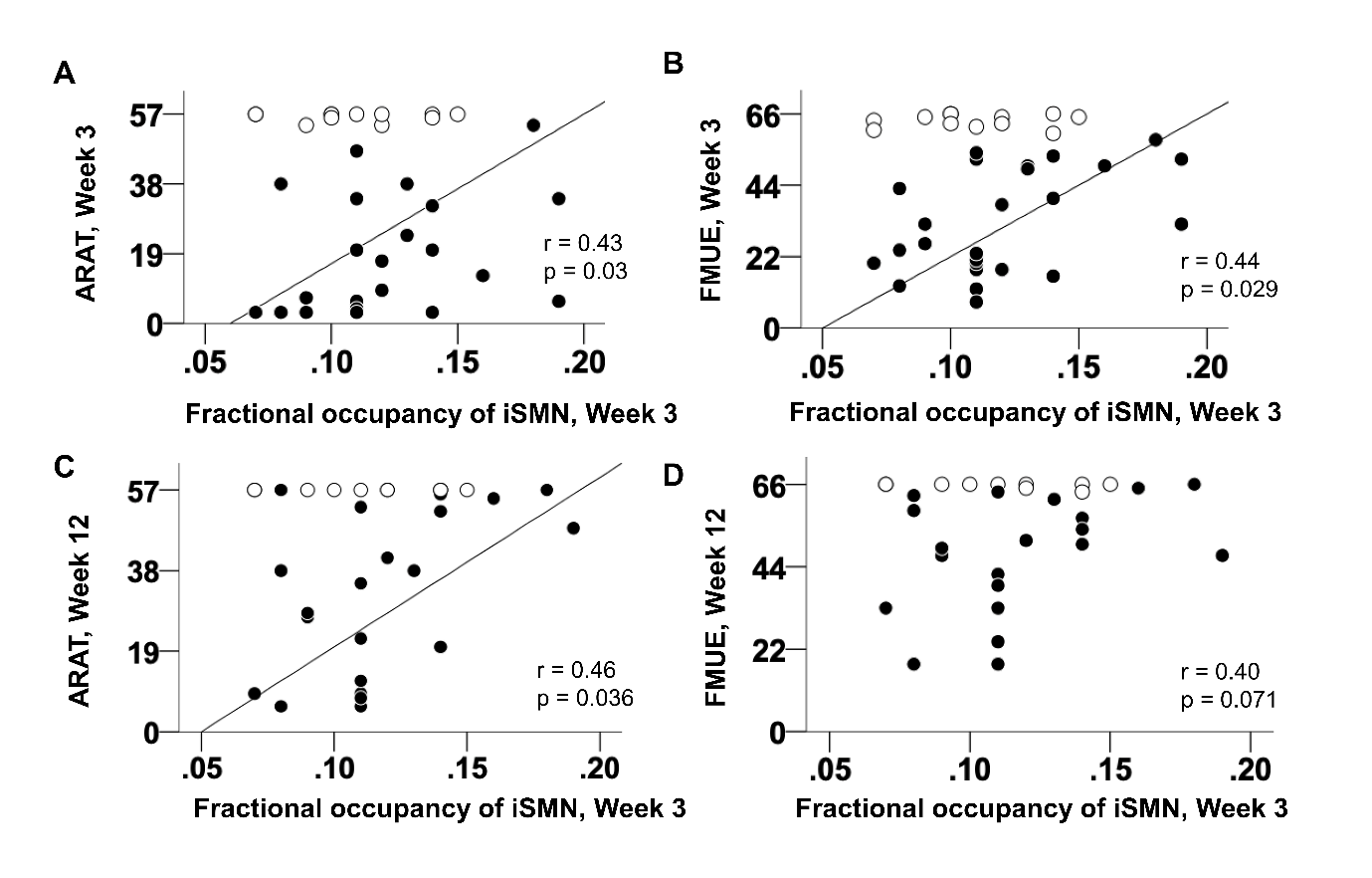 |
| --- |

## Supplementary Figure 1. Functionally correlated fractional occupancy in ipsilesional resting-state sensorimotor network

The mean fractional occupancy of ipsilesional sensorimotor network (iSMN) at week 3 after stroke in people with stroke showed a signficantly positive correlation with concurrent **(A)** action research arm test (ARAT) score and **(B)** Fugl-Meyer upper extremity (FM-UE) score, in those with initial moderate to severe hand paresis (n=25, dark circle ●) as defined by ARAT score <56 and FM-UE score <65 at stroke week 3. Such functional correlations were not seen in people with stroke with initial nearly fully recovered hand paresis (n=12, white circle **○**), defined by ARAT score of 56-57 or FM-UE score of 65-66 at stroke week 3. **(C)** The fractional occupancy of iSMN at stroke week 3 also positively correlated with ARAT at week 12 in those with initial moderate to severe hand paresis (n=21, dark circle ●), but not **(D)** FM-UE at week 12.
